# Role of signaling lymphocytic activation molecule family of receptors in the pathogenesis of rheumatoid arthritis: insights and application

**DOI:** 10.3389/fphar.2023.1306584

**Published:** 2023-11-06

**Authors:** Yixin Zheng, Jianan Zhao, Mi Zhou, Kai Wei, Ping Jiang, Lingxia Xu, Cen Chang, Yu Shan, Linshuai Xu, Yiming Shi, Steven J. Schrodi, Shicheng Guo, Dongyi He

**Affiliations:** ^1^ Department of Rheumatology, Shanghai Guanghua Hospital, Shanghai University of Traditional Chinese Medicine, Shanghai, China; ^2^ Guanghua Clinical Medical College, Shanghai University of Traditional Chinese Medicine, Shanghai, China; ^3^ Institute of Arthritis Research in Integrative Medicine, Shanghai Academy of Traditional Chinese Medicine, Shanghai, China; ^4^ Department of Rheumatology, Xiamen Hospital of Traditional Chinese Medicine, Xiamen, China; ^5^ Center for Human Genomics and Precision Medicine, University of Wisconsin-Madison, Madison, WI, United States; ^6^ Department of Medical Genetics, School of Medicine and Public Health, University of Wisconsin-Madison, Madison, WI, United States; ^7^ Arthritis Institute of Integrated Traditional and Western Medicine, Shanghai Chinese Medicine Research Institute, Shanghai, China

**Keywords:** rheumatoid arthritis, the signaling lymphocytic activation molecule family of receptors, immunity, inflammation, biomarker

## Abstract

Rheumatoid arthritis (RA) is an autoimmune disease characterized by chronic inflammation and joint damage. The signaling lymphocytic activation molecule (SLAMF) family of receptors are expressed on various hematopoietic and non-hematopoietic cells and can regulate both immune cell activation and cytokine production. Altered expression of certain SLAMF receptors contributes to aberrant immune responses in RA. In RA, SLAMF1 is upregulated on T cells and may promote inflammation by participating in immune cell-mediated responses. SLAMF2 and SLAMF4 are involved in regulating monocyte tumor necrosis factor production and promoting inflammation. SLAMF7 activates multiple inflammatory pathways in macrophages to drive inflammatory gene expression. SLAMF8 inhibition can reduce inflammation in RA by blocking ERK/MMPs signaling. Of note, there are differences in SLAMF receptor (SFR) expression between normal and arthritic joint tissues, suggesting a role as potential diagnostic biomarkers. This review summarizes recent advances on the roles of SLAMF receptors 1, 2, 4, 7, and 8 in RA pathogenesis. However, further research is needed to elucidate the mechanisms of SLAMF regulation of immune cells in RA. Understanding interactions between SLAMF receptors and immune cells will help identify selective strategies for targeting SLAMF signaling without compromising normal immunity. Overall, the SLAMF gene family holds promise as a target for precision medicine in RA, but additional investigation of the underlying immunological mechanisms is needed. Targeting SLAMF receptors presents opportunities for new diagnostic and therapeutic approaches to dampen damaging immune-mediated inflammation in RA.

## 1 Introduction

Rheumatoid arthritis (RA), characterized by chronic synovial inflammation, usually associated with joint swelling, tenderness, and pain (Smolen et al., 2016), is a an autoimmune disease that is a major source of disability and morbidity in society (Cross et al., 2014). RA is known to have a prevalence of approximately one percent within the general population, exhibiting a greater occurrence among females compared to males (Safiri et al., 2019). Delayed diagnosis and clinical intervention can dramatically increase joint damage. Thus, early diagnosis and treatment of inflammation is crucial to reducing further damage and preventing inflammation. RA is associated with a number of pathogenetic mechanisms, involving inflammation, metabolism, microorganisms, genetic components, immunity tolerance, epigenetic modifications, etc. (Okada et al., 2014; Lenz et al., 2015; Zhao et al., 2022a; Zhao et al., 2022b; Chang et al., 2022; Zhao et al., 2022c). Additionally, RA features elevated levels of autoreactive CD4^+^ T cells, pathogenic B cells, macrophages, inflammatory cytokines, and chemokines at the molecular and cellular levels (Jang et al., 2022). Recently, the signaling lymphocytic activation molecule family of receptors (SFRs) has been studied in relation to RA pathogenesis by participating in immune cell mediated inflammation (Isomäki et al., 1997; González-Alvaro et al., 2006; Li et al., 2022; Liu et al., 2022; Simmons et al., 2022). In addition, the signaling lymphocytic activation molecule family (SLAMF) genes were identified as susceptibility loci for RA. Overexpression of SLAMF genes was mostly found in the spleen, epstein-barr virus-transformed B lymphocytes, whole blood, lung and terminal ileum of the small intestine (Kwon et al., 2020). These studies point towards the importance of SLAMF genes in RA pathogenesis.

SFRs is involved in the regulation of T lymphocyte development and function, including the modulation of lymphocyte lysis activity, cytokine production, and other cellular processes. Furthermore, these receptors also govern the activation and generation of memory in B cells, as well as the functions of dendritic cells, macrophages, and other immune cell types (Detre et al., 2010; Howie et al., 2002; Réthi et al., 2006; Kis-Toth and Tsokos, 2014; O'Keeffe et al., 2015). SFRs consists of SLAMF1-9. SFRs are type I glycoproteins, composed of a N-terminal Ig variable region (V-region) lacking typical disulfide bonds and a C-terminal Ig constant 2-SET region (C-region) characterized by conserved cysteine. The cytoplasmic tail of typical SFRs contains multiple immune receptor tyrosine-based switching motifs (ITSM), while SLAMF2, SLAMF8, and SLAMF9 do not contain ITSM, making them considered atypical SFRs (Calpe et al., 2008). SFRs can recruit signaling molecules containing SH2 domains, such as SLAM related proteins (SAP) (Chan et al., 2003). The activation of SLAMF molecules phosphorylates ITSMs and provides binding sites for intracellular SAP or other SH2 containing enzymes, further transmitting different intracellular signals (Cocks et al., 1995; Howie et al., 2002; Wang et al., 2004; Dupré et al., 2005; Howie et al., 2005; Nanda et al., 2005; Nichols et al., 2005). SAP deficiency is associated with hereditary X-linked lymphoproliferative syndrome (Yan et al., 2007). It has been proposed that SFRs could be therapeutic targets for diseases related to inflammation and autoimmune responses (Dragovich and Mor, 2018). However, the specific mechanism of SFRs in RA remains unknown. This review summarizes recent progress of SFRs research on RA in a comprehensive manner, in order to discuss the potential SFRs as diagnostic and therapeutic targets in precision medicine.

## 2 SLAMF1- mediated inflammation in RA

A number of cells, including T cells, B cells, dendritic cells, and macrophages express SLAMF1 (Veillette, 2006). There is an association between SLAMF1 and the proliferation of B cells and the production of immunoglobulins (Punnonen et al., 1997). T cells, particularly CD4^+^T cells, express SLAMF1, demonstrating a low level of expression in immature cells, and a high level of expression in effector cells (Karampetsou et al., 2017). In addition to being upregulated after T and B cell activation, SLAMF1 may be a marker of several populations of white blood cells’ activation status (Punnonen et al., 1997; Castro et al., 1999).

In RA, the expression of SLAMF1 in synovial fluid and synovial tissue T cells from RA patients was significantly higher than that in peripheral blood T cells from the same patient or healthy controls (Isomäki et al., 1997). SLAMF1 expression was significantly reduced in CD4^+^T cells cultured with methotrexate compared to CD4^+^T cells cultured without methotrexate (Morita et al., 2006). SMLAF1 is a key biomarker for the development and progression of RA (12). In the collagen-induced arthritis (CIA) mouse model, immunohistochemical staining of joint tissue showed significant high expression of SLAMF1 in diseased joints. Flow cytometry analysis showed that SLAMF1 was present in cytotoxic T lymphocytes (CTLs), Th cells, natural killer cells (NKs), natural killer T cells (NKTs), dendritic cells (DCs), and M cells derived from CIA mice, with significantly increased expression (Li et al., 2022). Therefore, SLAMF1 may influence the pathogenesis of RA by participating in the inflammatory response mediated by these infiltrating immune cells. The ROC curve indicates that SLAMF1 has specific reference value in the diagnosis of RA patients (Li et al., 2022), suggesting that SLAMF1 may be an important biomarker for RA.

## 3 SLAMF2- and SLAMF4- mediated inflammation in RA

Several structural features of SLAMF2 are similar to those of other members of the Ig family. In this gene, there are four exons, one Ig variable (IgV) domain and one Ig constant (Ig-C2) domain, both with cysteine residues that allow disulfide bonds (Staunton et al., 1989). As compared to other members of the SLAMF, SLAMF2 is unique in that it does not have ITSM but can be activated when interacting with SLAMF4. Most hematopoietic cells, especially those presenting antigens, express SLAMF2 as a co-stimulatory molecule. SLAMF2 is involved in various innate and adaptive immune responses, including regulating granulocyte activity and inflammatory response, T cell activation and autoimmune response, and regulating CTL or NK cell function (McArdel et al., 2016).

SLAMF4 is considered an active receptor for NK cells, mediating non-MHC restricted killing effects (Assarsson et al., 2004). SLAMF4 can express on NK cells, most CD8^+^T cells, and monocytes. Human and mouse Ig-V and Ig-C2 domains have 40% sequence homology, with 8 potential N-terminal glycosylation sites (Kumaresan and Mathew, 2000). T cells and NK cells can be activated or inhibited by SLAMF4. When sufficient amounts of SAP are present on target cells, SLAMF4-SLAMF2 interactions promote NK or T cell activation. However, in the absence of SAP on the target cells, SLAMF4 produces an inhibitory signal (Farhangnia et al., 2023).

A significant correlation exists between SLAMF2 and the infiltration of five types of immune cells, namely, plasma cells, CD4^+^ naive T cells, CD4^+^ activated memory T cells, gamma delta T cells, and activated NK cells. The differences of SLAMF2 expression in these immune cells in the normal and RA samples are significant (Sun et al., 2022). The expression of SLAMF4 in RA serum significantly increases when compared to healthy controls, indicating that it may contribute to RA pathogenesis (Han et al., 2022). Matsui et al. (1990) analyzed the gene encoding SLAMF2 and discovered that SLAMF2 is a possible genetic marker for the manifestation of RA. Suzuki et al. (2008) discovered that SLAMF4 is a genetic risk factor for RA and may play a role in the common autoimmune processes of RA. Fasth et al. (2010) found that compared to healthy controls, the expression of various NK receptors, including SLAMF4, in peripheral blood CD4^+^CD28^−^T cells of RA patients enhanced the immune response. CD4^+^CD28^−^ T cells can be characterized as extensively differentiated effector memory CD4^+^T cells. RA patients’ blood and synovial fluid contain these cells, which exacerbate inflammatory responses. SLAMF4 can lead to disease progression by enhancing the function of these cells. Apart from that, among monocytic cells, SLAMF2 and SLAMF4 play a role in regulating tumor necrosis factor (TNF) production. Blocking SLAMF2 and SLAMF4 reversed this phenotype, although the specific mechanism is still unclear (González-Alvaro et al., 2006). Based on these results, it appears SLAMF2 and SLAMF4 affect the development of RA through their pro-inflammatory effects.

## 4 The role of SLAMF5 in immune regulation and RA

SLAMF5 is a single-chain glycoprotein on the cell surface, with 199 amino acids in its extracellular segment and four potential N-terminal glycosylation sites. The transmembrane region contains 25 amino acids, the intracellular region contains 83 amino acids and 4 tyrosines, with two tyrosines embedded in ITSM (Martin et al., 2001; Zaiss et al., 2003). SLAMF5 mainly expresses on B cells, T cells, platelets, monocytes, DCs, and early hematopoietic stem cells (Falco et al., 2004). SLAMF5 widely expresses in most immune cell subpopulations and is an isotropic adhesion molecule. Its signal transduction can activate or inhibit white blood cell function based on cell type. The signaling mediated by SLAMF5 regulates a variety of immune functions. This includes cytokine secretion by T cells, cytotoxicity by natural killer cells, monocyte activation, autophagy, and homologous T:B interactions within germinal centers. According to recent studies, changes in SLAMF5 are related to autoimmune diseases, such as X-linked lymphoproliferative syndrome, systemic lupus erythematosus, and RA (Cuenca et al., 2019). Researchers found that PD-1hiCXCR5CD4, a novel CD4 T peripheral helper cell population, infiltrated inflamed tissue in RA and provided B cell helper functions. A high level of SAP, SLAMF1, SLAMF5, and SLAMF6 is expressed in these cells. Plasma cell differentiation and IgG production were completely abrogated by antibody blockade of SLAMF5 (Rao et al., 2017). Furthermore, individuals of European ancestry might be able to predict etanercept treatment response based on the genotype or expression of SLAMF5. A positive relationship between SLAMF5 expression and treatment response is predicted by genetic and expression data. The higher expression is associated with improved response (Cui et al., 2013).

## 5 The role of SLAMF6 in immune regulation and RA

Human SLAMF6 has two extracellular Ig domains with seven potential N-glycosylation sites. The cytoplasm tail contains 83 amino acids and 3 tyrosine residues, with 4 embedded in the ITSM domain (Cao et al., 2006). SLAMF6 mainly expresses in human and mouse lymphatic organs, with high expression in the spleen, thymus, and lymph nodes, and relatively low expression in bone marrow, lungs, and liver (Eissmann and Watzl, 2006). As a positive and negative regulator of immunity, SLAMF6 is highly expressed in activated T and B cells. SLAMF6 also expresses in NK cells (Fraser et al., 2002; Wang et al., 2015). Engagement of SLAMF6 influences TCR activation on a variety of levels, including cytokine secretion, proliferation, and cellular adhesion via Rap1 activation (Dragovich et al., 2019).

Kwon YC et al. identified *SLAMF6* as new RA-associated loci, and found that A variant of *SLAMF6* (rs148363003) interacts with other SLAMF coding genes including *SLAMF1*, *SLAMF2*, *SLAMF3*, *SLAMF5*, *SLAMF7* on chromosome 1. The overexpression of *SLAMF6* and other interacting SLAMF genes was primarily found in spleens, EBV-transformed B lymphocytes, whole blood, lungs, and terminal ileums of the small intestine. Which underscored the importance of the immune system as well as non-immune tissues for the development of RA (Kwon et al., 2020). Xia et al. (2022) analyzed the expression of *SLAMF6* gene in synovial tissue of RA patients and in the control group. The expression of *SLAMF6* was significantly higher in RA patients than that in the control group. The expression of *SLAMF6* in patients with genotype CC rs148363003 was significantly higher than that in patients with genotype TT. Due to the significantly higher frequency of genotype CC in patients compared to the normal control group, the variant allele C may increase the risk of RA through *SLAMF6* regulation. RA patients with high activity levels expressed more *SLAMF6* than RA patients with low activity levels. Additionally, serum RF was significantly correlated with *SLAMF6* expression. Therefore, SLAMF6 may serve as a regulatory gene and predict the progression of RA.

## 6 SLAMF7 exhibited therapeutic potential for RA patients

SLAMF7 possesses a relative molecular weight of 66 kDa. Within its ITSMs, two conserved tyrosine residues, namely, Y281 and Y261, play pivotal roles in the activation and inhibition signaling pathways of SLAMF7(59). SLAMF7 is consistently expressed at low levels in various immune cells in the human body, including NK cells, CD4^+^T cells, CD8^+^T cells, some B cells, macrophages, and DCs (Wang et al., 2004; Lee et al., 2007; van Driel et al., 2016; Chen et al., 2017). However, the expression level of SLAMF7 in NK cells is higher than that of other immune cells, and it continues to be expressed throughout the development of NK cells, participating in regulating the lysis function of NK cells towards target cells (Chen et al., 2016). Research has found that as CD8^+^T cells differentiate, the expression of SLAMF7 increases, and most effector memory and terminal differentiation effector memory CD8^+^T cells express SLAMF7 (Bae et al., 2012).

RA patients have already shown potential therapeutic benefit from SLAMF7. Simmons et al. (2022) discovered upregulation of SLAMF7 on macrophages from inflamed synovial tissue. The SLAMF7 levels were twice as high on synovial fluid macrophages from RA patients when compared to osteoarthritis (OA) patients. Engagement of SLAMF7 with recombinant SLAMF7 protein resulted in doubling of phosphorylation of extracellular regulated protein kinases (ERK) and more than four times more phosphorylation of NF-κB P65 at the time points tested. Also, more than ten times higher phosphorylation of mitogen-activated protein kinase (MAPK) P38 and an almost three-fold increase in AKT phosphorylation was detected, suggesting SLAMF7 as a receptor that activates multiple pathways that reprogram macrophage metabolism to drive inflammatory gene expression. Secreted TNF-α levels and interleukin-6 (IL-6) levels increased after stimulation. The induction of CCL3, CXCL, and CXCL8 after SLAMF7 engagement was confirmed by real-time PCR analysis. By targeting SLAMF7 and downstream pathways, inflammation caused by macrophages may be blocked while maintaining moderate immune surveillance and homeostatic macrophage functions. Another study (Woo et al., 2013) demonstrated that in RA synovium, SLAM7 was found to be highly expressed on CD20 plasmablast and plasma cell populations, leading to investigations of its therapeutic potential. Some patients have been unable to benefit from the use of anti-CD20 monoclonal antibodies, which deplete B cells from the circulation (Edwards et al., 2004). They have persistent CD20-negative plasmablasts and plasma cells (Owczarczyk et al., 2011). SLAMF7(67) is strongly expressed in these cells (Hsi et al., 2008), so a humanized antibody called PDL241 was developed to target it. Plasmablasts and plasma cells are killed by PDL241, but B cells are not affected, inhibiting immunoglobulin production in a Fc-dependent manner (Woo et al., 2013). Additionally, rhesus monkeys treated with PDL214 had a reduction in IgG and IgM antibodies that reduced joint-related disease parameters (Woo et al., 2013).

## 7 SLAMF8-mediated signaling via ERK/MMPs pathway activation promotes inflammation in RA

Based on a cloned human MLR library, SLAMF8 is a type I transmembrane protein that has three potential N-glycosylation sites, including an extracellular domain of 212 amino acids, a transmembrane domain of 21 amino acids, and a short cytoplasmic tail of 31 amino acids. In the extracellular domain, there are two typical Ig-like domains, an N-terminal fold that is similar to the IgV fold without conserving disulfide bonds, and a membrane-proximal fold that is similar to the C2 fold. The mouse homologous strain exhibits 75% identity with human SLAMF8 at the amino acid level (Kingsbury et al., 2001). SLAMF8 expresses on APCs, DCs, and activated monocytes (Kingsbury et al., 2001). Through B cell receptors, it also regulates signaling (Shachar et al., 2019). Cancer cells proliferate more quickly when SLAMF8 is overexpressed in anaplastic large cell lymphoma (Sugimoto et al., 2020; Zhang et al., 2021). Glioma progression, poor prognosis, and chemotherapy resistance are also associated with SLAMF8, making it a potential therapeutic target (Zou et al., 2019). Researchers have discovered that SLAMF8 is highly expressed in gastric cancer patients’ serum. Moreover, it can be used to diagnose and prognosis cancer at an early stage (Wu et al., 2019). Additionally, SLAMF8 acts as a tyrosine kinase inhibitor and contributes to carcinogenic KIT signaling through RAS/RAF/ERK and human tumor mast cell proliferation through SHP-2 binding (Sugimoto et al., 2018). In RA, the ERK pathway can increase the expression of matrix metalloproteinase-1 (MMP-1) and matrix metalloproteinase-13 (MMP-13) (Agere et al., 2017).

The overexpression of SLAMF8 is associated with disease activity and increased inflammation in RA (Chen et al., 2022). found that the HBEGF^+^ macrophages especially express SLAMF8. The expression was upregulated under the RA state, whereas it was decreased after triple DMARD treatment (methotrexate, sulfasalazine, and hydroxychloroquine), suggesting that SLAMF8 is involved in the pathogenies of RA. Researchers have identified the *SLAMF8* gene as a differentially expressed gene in RA samples analyzed from gene expression databases and synovial tissue samples collected from RA patients (Qin et al., 2022). In RA, *SLAMF8* expression is significantly higher than in OA, and it correlates positively with disease activity and inflammation. Animal experiments found that *SLAMF8* is upregulated in the RA mouse model (Qin et al., 2022). Further knockout of *SLAMF8* revealed inhibition of TLR4 expression, blocking NF-κB signaling in the RA model, ultimately alleviating synovial hyperplasia and arthritis in mice (Liu et al., 2022). discovered that after TNF-α treatment, the expression of *SLAMF8* mRNA and protein in MH7A and HFLS-RA cell lines increased in a time-dependent manner, while *SLAMF8* knocking significantly reduced the TNF-α-induced pro-inflammatory response in MH7A and HFLS-RA cells, including proliferation, invasion and migration in the cells. At the same time, silencing SLAMF8 also significantly suppresses p-ERK, MMP-1, and MMP-13 expression.

## 8 The role of SLAMF3 and SLAMF9 in RA remains unknown

The typical members of SLAMF3 have a repeated IgV-like domain and a C2 domain, which makes four extracellular domains (Sandrin et al., 1996). SLAMF3 expresses on B and T lymphocytes (de la Fuente et al., 2001), and significantly affects the differentiation, expansion, and function of T cells (Chatterjee et al., 2012a). Induction of Th17 phenotypes and expression of IL-17A may be mediated by SLAMF3 and SLAMF6 co-stimulatory molecules (Chatterjee et al., 2012b). The gene position of SLAMF9 is close to SLAMF8, and its mature protein includes an extracellular segment composed of 289 amino acids, a transmembrane region composed of 20 amino acids, and a cytoplasmic tail end composed of only 32 amino acids. Its extracellular domain includes two potential N-glycosylation sites, with 45% and 41% homology with the amino acid sequences of SLAMF3 and SLAMF5, respectively. It is a homologous recognition receptor, as the tail end of SLAMF9 cytoplasm does not have an ITSM domain, therefore it is generally believed that it does not have the function of transmitting signals (Volkova et al., 2014). *SLAMF9* mRNA has been found to be expressed in human monocytes, T, B, and DC cells (Calpe et al., 2008). At present, no research has been found on the relationship between SLAMF3, SLAMF9, and RA.

## 9 Discussion

**FIGURE 1:**
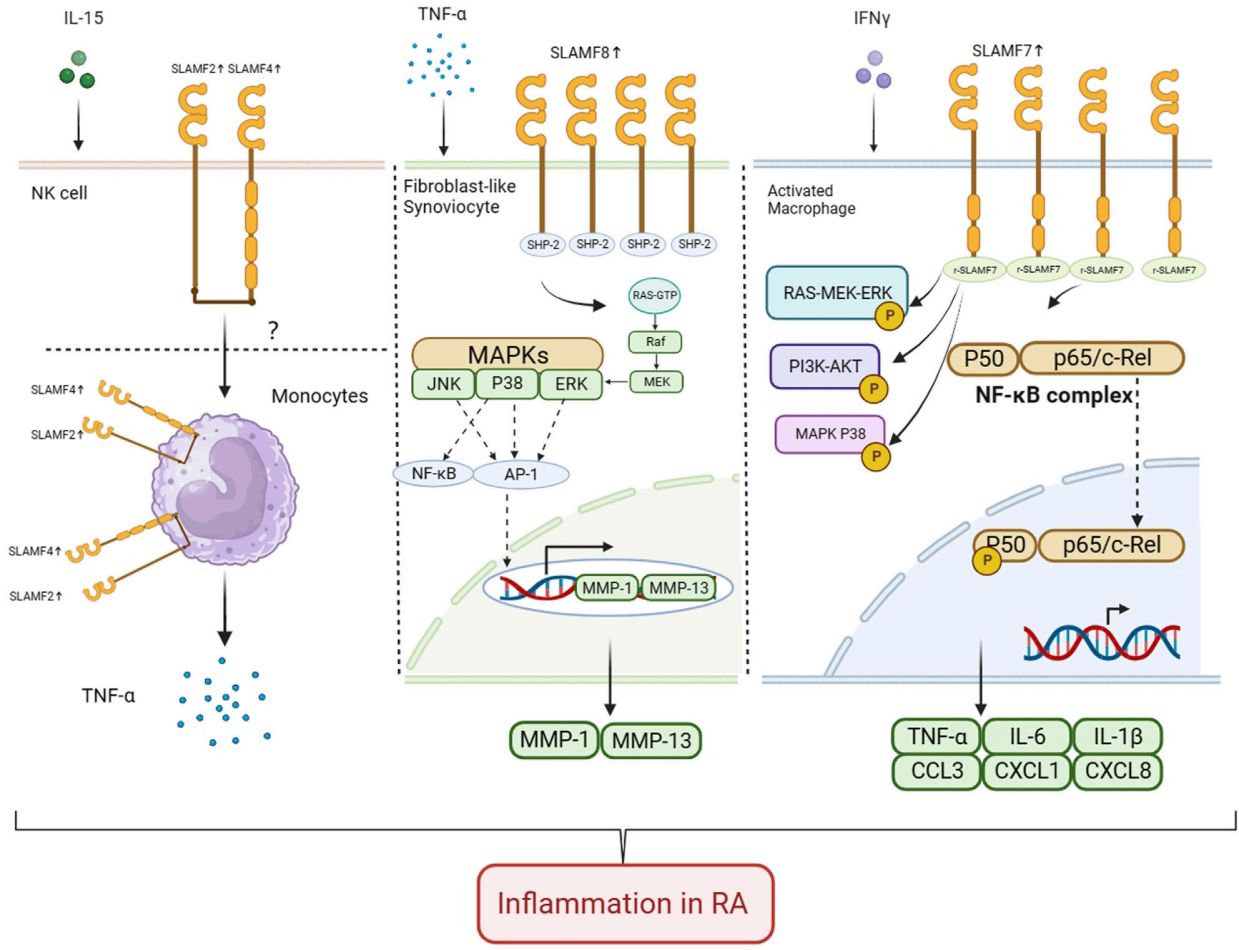
Role of SLAMF2, SLAMF4, SLAMF7, and SLAMF8 in pathology and progression of RA In RA, IL-15 enhances the expression of SLAMF2 in NK cells. SLAMF4 and SLAMF2 are involved in intercellular contact regulation of monocyte TNF production, but the specific mechanism is not yet clear. The expression of SLAMF8 is upregulated on HFLS-RA cells. SLAMF8 promotes proliferation and migration of synovioblast in RA. SLAMF8 knockdown significantly attenuates TNF-α-induced pro-inflammatory response in MH7A and HFLS-RA cells, and the expression of p-ERK, MMP-1 and MMP-13 is significantly inhibited when SLAMF8 is silenced. The expression of SLAMF7 on macrophages from inflamed synovial tissue is upregulated. Engagement of SLAMF7 with recombinant SLAMF7 protein resulted in doubling of phosphorylation of ERK and more than four times more phosphorylation of NF-κB P65 at the time points tested. Also, more than ten times higher phosphorylation of MAPK P38 and an almost three-fold increase in AKT phosphorylation was detected. Secreted TNF-α levels and IL-6 levels increased after stimulation. Real-time PCR analysis confirmed induction of CCL3, CXCL, and CXCL8 after SLAMF7 engagement (Created with BioRender.com).

Our review discusses the physiological function, cellular expression, and potential role of SFRs in RA (Table 1). In recent years, research has found that SFRs can promote inflammation and affect the progression of RA in different ways (Figure 1). RA is thought to be primarily caused by abnormal accumulation of fibroblast-like synoviocytes (FLSs), macrophages, and other immune cells in synovial joints (Korb-Pap et al., 2012). Upregulation of the SLAMF gene can be observed in T cells, macrophages, and fibroblasts in the synovium and peripheral blood of RA patients. The expression of SLAMF1 and SLAMF4 is increased in T cells of synovium and peripheral blood in RA patients, respectively.

**TABLE 1:** The physiological function, cellular expression, and potential role of the SLAMF in RA.

SLAMF	Cellular expression in RA	Function in RA
SLAMF1	T cells (human) CTL, Th cells, NK cells, NKT cells (mice)	Compared to peripheral blood T cells from the same patient or healthy volunteers, SLAMF1 expression levels in synovial fluid and synovial tissue T cells of RA patients were significantly upregulated. In the CIA mouse model, immunohistochemical staining of joint tissue showed significant high expression of SLAMF1 in diseased joints. SLAMF1 was present in CTL, Th cells, NK cells, NKT cells, etc. derived from CIA mice. Therefore, SLAMF1 may affect the pathogenesis of RA by participating in the inflammatory response mediated by these infiltrating immune cells.
SLAMF2	NK cells Monocytes	IL-15 enhances the expression of SLAMF2 in NK cells. In RA, SLAMF4 and SLAMF2 are involved in intercellular contact regulation of monocyte TNF production, but the specific mechanism is not yet clear.
SLAMF4	NK cells Monocytes CD4^+^ T cells	In RA, SLAMF4 and SLAMF2 are involved in intercellular contact regulation of monocyte TNF production, but the specific mechanism is not yet clear. Also, compared with healthy controls, the expression of SLAMF4 in peripheral blood CD4+CD28−T cells of RA patients is upregulated.
SLAMF7	Macrophages	Engagement of SLAMF7 with recombinant SLAMF7 protein resulted in doubling of phosphorylation of ERK and more than four times more phosphorylation of NF-κB P65 at the time points tested. Also, more than ten times higher phosphorylation of MAPK P38 and an almost three-fold increase in AKT phosphorylation was detected. Secreted TNF-α levels and IL-6 levels increased after stimulation. Real-time PCR analysis confirmed induction of CCL3, CXCL, and CXCL8 after SLAMF7 engagement.
SLAMF8	HFLS-RA cells	After TNF-α treatment, the expression of SLAMF8 mRNA and protein in MH7A and HFLS-RA cell lines increased in a time-dependent manner, while SLAMF8 knocking significantly reduced the TNF-α-induced pro-inflammatory response in MH7A and HFLS-RA cells, including proliferation, invasion and migration in the cells. At the same time, when SLAMF8 is silent, the expression of p-ERK, MMP-1 and MMP-13 is also significantly suppressed.

RA is characterized by long-term chronic synovitis, cartilage necrosis, and eventually joint damage (Smolen et al., 2016). Treg cells affect autoimmune responses (Kamradt and Mitchison, 2001). By reducing the number or function of these cells, the immune cascade is amplified, raising the levels of various cytokines quickly, and activates macrophages in the synovium of bones and joints to produce inflammatory cytokines including IL-1, IL-6, and IL-8 (Jiang et al., 2021). Inhibiting the excessive activation of T cells is considered one of the therapeutic targets for RA. T cell activation relies on the first and second signals, with the second signal being a co stimulatory molecule on the surface of antigen presenting cells. SLAMF1 has T-cell co-stimulation function (Cocks et al., 1995). The elevation of SLAMF1 in T cells in RA patients may affect the progression of RA through the activation of T cells. Due to the non-specific and intervenable nature of the second signal, regulating the second signal to enhance or weaken T cell activity is currently an important drug development target, thus targeting SLAMF1 has the potential to treat RA.

SLAMF7 upregulates on macrophages from inflamed synovial tissue (Simmons et al., 2022). In RA synovium, activated macrophages also produce enzymes that contribute to tissue damage (Culemann et al., 2019). CCL- and CXCL-chemokines, and TNF-α, IL-6, IL-1β or IL-23 promote and sustain inflammation by recruiting polymorphonuclear cells, T cells, B cells, and monocytes (Elemam et al., 2020). Macrophages also make angiogenic factors, which contribute to the hypervascularization seen in RA (Maruotti et al., 2013). Depending on the microenvironment, macrophages can also differentiate directly into mature osteoclasts (Pereira et al., 2018). Due to their consistent production of MMPs (Huang et al., 2012), inflammatory macrophages are an important contributor to the turnover of connective tissue and erosion of articular surfaces observed in RA (Rose and Kooyman, 2016).

SLAMF8 is upregulated in fibroblasts of synovial tissue. The main pathological changes of RA are synovitis and joint destruction. This process is mainly mediated by RA synovial fibroblasts (FLS). RA-FLS can secrete a large amount of pro-inflammatory cytokines to exacerbate local inflammatory reactions in joints, and secrete matrix metalloproteinases and cathepsin to degrade cartilage matrix. The abnormal proliferation of RA-FLS cells not only participates in the formation of “pannus,” but also multiplies the release of inflammatory and invasive factors, ultimately accelerating the occurrence and development of joint synovitis and bone destruction (Bartok and Firestein, 2010). When *SLAMF8* was knocked down, TNF-α-induced proinflammatory responses in MH7A and HFLS-RA cells were significantly reduced, and silencing *SLAMF8* significantly inhibited the expression of p-ERK, MMP-1, and MMP-13 (Liu et al., 2022). Correlations of serum MMP-1 and MMP-3 levels with the degree of disease activity predict the progression of RA in terms of joint damage (Hattori et al., 2019; Tuncer et al., 2019). The activity of ERK is correlated with RA, with ERK inhibitors weakening antigen-specific T-cell activation (Ohori, 2008), which plays an important role as RA develops. Therefore, targeted inhibition of SLAMF8 can alleviate inflammation in RA by blocking the ERK/MMP signaling pathway.

In summary, this review provides current understanding of SLAMF genes’ roles in RA pathogenesis. The study of SFRs has the potential to provide important insights into the mechanisms of RA, and to identify new therapeutic targets for precision medicine. There is a need for more research into how SLAMF receptors regulate immune cell activation and function, and the interactions between immune cells in the context of RA. The study of SLAMF gene family presents a unique opportunity to improve our understanding of the pathogenesis of RA, and to develop new therapeutic strategies for the treatment.

## Abbreviations

Rheumatoid arthritis, RA; signaling lymphocytic activation molecule, SLAMF; Cluster of Differentiation, CD; Immune receptor tyrosine-based switching motifs, ITSM; SLAM associated protein, SAP; Collagen-induced arthritis, CIA; Cytotoxic T lymphocytes, CTLs; Th cells, natural killer cells, NKs; Natural killer T cells, NKTs; Dendritic cells, DCs; Antigen presenting cells, APCs; Cytotoxic lymphocytes, CLs; Cytotoxic T lymphocytes, CTLs; Interleukin, L; Toll-like receptor, TLR; Tumor necrosis factor, TNF; Rheumatoid factor, RF; Osteoarthritis, OA; Extracellular regulated protein kinases, RK; Mitogen-activated protein kinase, MAPK; Receiver operating characteristic curve, ROC; Metalloproteinase, MMP.

## References

[B1] Agere S. A. Akhtar N. Watson J. M. Ahmed S. (2017). RANTES/CCL5 induces collagen degradation by activating MMP-1 and MMP-13 expression in human rheumatoid arthritis synovial fibroblasts. Front. Immunol. 8, 1341. 10.3389/fimmu.2017.01341 29093715 PMC5651228

[B2] Assarsson E. Kambayashi T. Schatzle J. D. Cramer S. O. von Bonin A. Jensen P. E. (2004). NK cells stimulate proliferation of T and NK cells through 2B4/CD48 interactions. J. Immunol. 173 (1), 174–180. 10.4049/jimmunol.173.1.174 15210772

[B3] Bae J. Song W. Smith R. Daley J. Tai Y. T. Anderson K. C. (2012). A novel immunogenic CS1-specific peptide inducing antigen-specific cytotoxic T lymphocytes targeting multiple myeloma. Br. J. Haematol. 157 (6), 687–701. 10.1111/j.1365-2141.2012.09111.x 22533610 PMC3819168

[B4] Bartok B. Firestein G. S. (2010). Fibroblast-like synoviocytes: key effector cells in rheumatoid arthritis. Immunol. Rev. 233 (1), 233–255. 10.1111/j.0105-2896.2009.00859.x 20193003 PMC2913689

[B5] Calpe S. Wang N. Romero X. Berger S. B. Lanyi A. Engel P. (2008). The SLAM and SAP gene families control innate and adaptive immune responses. Adv. Immunol. 97, 177–250. 10.1016/s0065-2776(08)00004-7 18501771

[B6] Cao E. Ramagopal U. A. Fedorov A. Fedorov E. Yan Q. Lary J. W. (2006). NTB-A receptor crystal structure: insights into homophilic interactions in the signaling lymphocytic activation molecule receptor family. Immunity 25 (4), 559–570. 10.1016/j.immuni.2006.06.020 17045824

[B7] Castro A. G. Hauser T. M. Cocks B. G. Abrams J. Zurawski S. Churakova T. (1999). Molecular and functional characterization of mouse signaling lymphocytic activation molecule (SLAM): differential expression and responsiveness in Th1 and Th2 cells. J. Immunol. 163 (11), 5860–5870. 10.4049/jimmunol.163.11.5860 10570270

[B8] Chan B. Lanyi A. Song H. K. Griesbach J. Simarro-Grande M. Poy F. (2003). SAP couples Fyn to SLAM immune receptors. Nat. Cell. Biol. 5 (2), 155–160. 10.1038/ncb920 12545174

[B9] Chang C. Xu L. Zhang R. Jin Y. Jiang P. Wei K. (2022). MicroRNA-mediated epigenetic regulation of rheumatoid arthritis susceptibility and pathogenesis. Front. Immunol. 13, 838884. 10.3389/fimmu.2022.838884 35401568 PMC8987113

[B10] Chatterjee M. Hedrich C. M. Rauen T. Ioannidis C. Terhorst C. Tsokos G. C. (2012b). CD3-T cell receptor co-stimulation through SLAMF3 and SLAMF6 receptors enhances RORγt recruitment to the IL17A promoter in human T lymphocytes. J. Biol. Chem. 287 (45), 38168–38177. 10.1074/jbc.M112.415067 22989874 PMC3488086

[B11] Chatterjee M. Rauen T. Kis-Toth K. Kyttaris V. C. Hedrich C. M. Terhorst C. (2012a). Increased expression of SLAM receptors SLAMF3 and SLAMF6 in systemic lupus erythematosus T lymphocytes promotes Th17 differentiation. J. Immunol. 188 (3), 1206–1212. 10.4049/jimmunol.1102773 22184727 PMC3262878

[B12] Chen J. Zhong M. C. Guo H. Davidson D. Mishel S. Lu Y. (2017). SLAMF7 is critical for phagocytosis of haematopoietic tumour cells via Mac-1 integrin. Nature 544 (7651), 493–497. 10.1038/nature22076 28424516 PMC5565268

[B13] Chen N. Fan B. He Z. Yu X. Wang J. (2022). Identification of HBEGF+ fibroblasts in the remission of rheumatoid arthritis by integrating single-cell RNA sequencing datasets and bulk RNA sequencing datasets. Arthritis Res. Ther. 24 (1), 215. 10.1186/s13075-022-02902-x 36068607 PMC9446562

[B14] Chen S. Yang M. Du J. Li D. Li Z. Cai C. (2016). The self-specific activation receptor SLAM family is critical for NK cell education. Immunity 45 (2), 292–304. 10.1016/j.immuni.2016.07.013 27521267

[B15] Cocks B. G. Chang C. C. Carballido J. M. Yssel H. de Vries J. E. Aversa G. (1995). A novel receptor involved in T-cell activation. Nature 376 (6537), 260–263. 10.1038/376260a0 7617038

[B16] Cross M. Smith E. Hoy D. Carmona L. Wolfe F. Vos T. (2014). The global burden of rheumatoid arthritis: estimates from the global burden of disease 2010 study. Ann. Rheum. Dis. 73 (7), 1316–1322. 10.1136/annrheumdis-2013-204627 24550173

[B17] Cuenca M. Sintes J. Lányi Á. Engel P. (2019). CD84 cell surface signaling molecule: an emerging biomarker and target for cancer and autoimmune disorders. Clin. Immunol. 204, 43–49. 10.1016/j.clim.2018.10.017 30522694

[B18] Cui J. Stahl E. A. Saevarsdottir S. Miceli C. Diogo D. Trynka G. (2013). Genome-wide association study and gene expression analysis identifies CD84 as a predictor of response to etanercept therapy in rheumatoid arthritis. PLoS Genet. 9 (3), e1003394. 10.1371/journal.pgen.1003394 23555300 PMC3610685

[B19] Culemann S. Grüneboom A. Krönke G. (2019). Origin and function of synovial macrophage subsets during inflammatory joint disease. Adv. Immunol. 143, 75–98. 10.1016/bs.ai.2019.08.006 31607368

[B20] de la Fuente M. A. Tovar V. Villamor N. Zapater N. Pizcueta P. Campo E. (2001). Molecular characterization and expression of a novel human leukocyte cell-surface marker homologous to mouse Ly-9. Blood 97 (11), 3513–3520. 10.1182/blood.v97.11.3513 11369645

[B21] Detre C. Keszei M. Romero X. Tsokos G. C. Terhorst C. (2010). SLAM family receptors and the SLAM-associated protein (SAP) modulate T cell functions. Semin. Immunopathol. 32 (2), 157–171. 10.1007/s00281-009-0193-0 20146065 PMC2868096

[B22] Dragovich M. A. Adam K. Strazza M. Tocheva A. S. Peled M. Mor A. (2019). SLAMF6 clustering is required to augment T cell activation. PLoS One 14 (6), e0218109. 10.1371/journal.pone.0218109 31199820 PMC6568412

[B23] Dragovich M. A. Mor A. (2018). The SLAM family receptors: potential therapeutic targets for inflammatory and autoimmune diseases. Autoimmun. Rev. 17 (7), 674–682. 10.1016/j.autrev.2018.01.018 29729453 PMC6508580

[B24] Dupré L. Andolfi G. Tangye S. G. Clementi R. Locatelli F. Aricò M. (2005). SAP controls the cytolytic activity of CD8+ T cells against EBV-infected cells. Blood 105 (11), 4383–4389. 10.1182/blood-2004-08-3269 15677558

[B25] Edwards J. C. Szczepanski L. Szechinski J. Filipowicz-Sosnowska A. Emery P. Close D. R. (2004). Efficacy of B-cell-targeted therapy with rituximab in patients with rheumatoid arthritis. N. Engl. J. Med. 350 (25), 2572–2581. 10.1056/NEJMoa032534 15201414

[B26] Eissmann P. Watzl C. (2006). Molecular analysis of NTB-A signaling: a role for EAT-2 in NTB-A-mediated activation of human NK cells. J. Immunol. 177 (5), 3170–3177. 10.4049/jimmunol.177.5.3170 16920955

[B27] Elemam N. M. Hannawi S. Maghazachi A. A. (2020). Role of chemokines and chemokine receptors in rheumatoid arthritis. ImmunoTargets Ther. 9, 43–56. 10.2147/ITT.S243636 32211348 PMC7074856

[B28] Falco M. Marcenaro E. Romeo E. Bellora F. Marras D. Vély F. (2004). Homophilic interaction of NTBA, a member of the CD2 molecular family: induction of cytotoxicity and cytokine release in human NK cells. Eur. J. Immunol. 34 (6), 1663–1672. 10.1002/eji.200424886 15162436

[B29] Farhangnia P. Ghomi S. M. Mollazadehghomi S. Nickho H. Akbarpour M. Delbandi A. A. (2023). SLAM-family receptors come of age as a potential molecular target in cancer immunotherapy. Front. Immunol. 14, 1174138. 10.3389/fimmu.2023.1174138 37251372 PMC10213746

[B30] Fasth A. E. Björkström N. K. Anthoni M. Malmberg K. J. Malmström V. (2010). Activating NK-cell receptors co-stimulate CD4(+)CD28(-) T cells in patients with rheumatoid arthritis. Eur. J. Immunol. 40 (2), 378–387. 10.1002/eji.200939399 19904767

[B31] Fraser C. C. Howie D. Morra M. Qiu Y. Murphy C. Shen Q. (2002). Identification and characterization of SF2000 and SF2001, two new members of the immune receptor SLAM/CD2 family. Immunogenetics 53 (10-11), 843–850. 10.1007/s00251-001-0415-7 11862385

[B32] González-Alvaro I. Domínguez-Jiménez C. Ortiz A. M. Núñez-González V. Roda-Navarro P. Fernández-Ruiz E. (2006). Interleukin-15 and interferon-gamma participate in the cross-talk between natural killer and monocytic cells required for tumour necrosis factor production. Arthritis Res. Ther. 8 (4), R88. 10.1186/ar1955 16684368 PMC1779407

[B33] Han Y. Lu X. Lai W. Liang R. Yang M. Ouyang Q. (2022). Identification of serological biomarkers for diagnosis of rheumatoid arthritis using a protein array-based approach. Nan Fang. Yi Ke Da Xue Xue Bao 42 (5), 733–739. 10.12122/j.issn.1673-4254.2022.05.15 35673918 PMC9178634

[B34] Hattori Y. Kida D. Kaneko A. (2019). Normal serum matrix metalloproteinase-3 levels can be used to predict clinical remission and normal physical function in patients with rheumatoid arthritis. Clin. Rheumatol. 38 (1), 181–187. 10.1007/s10067-017-3829-9 28940139

[B35] Howie D. Laroux F. S. Morra M. Satoskar A. R. Rosas L. E. Faubion W. A. (2005). Cutting edge: the SLAM family receptor Ly108 controls T cell and neutrophil functions. J. Immunol. 174 (10), 5931–5935. 10.4049/jimmunol.174.10.5931 15879084

[B36] Howie D. Okamoto S. Rietdijk S. Clarke K. Wang N. Gullo C. (2002). The role of SAP in murine CD150 (SLAM)-mediated T-cell proliferation and interferon gamma production. Blood 100 (8), 2899–2907. 10.1182/blood-2002-02-0445 12351401

[B37] Hsi E. D. Steinle R. Balasa B. Szmania S. Draksharapu A. Shum B. P. (2008). CS1, a potential new therapeutic antibody target for the treatment of multiple myeloma. Clin. Cancer Res. 14 (9), 2775–2784. 10.1158/1078-0432.Ccr-07-4246 18451245 PMC4433038

[B38] Huang W. C. Sala-Newby G. B. Susana A. Johnson J. L. Newby A. C. (2012). Classical macrophage activation up-regulates several matrix metalloproteinases through mitogen activated protein kinases and nuclear factor-κB. PLoS One 7, e42507. 10.1371/journal.pone.0042507 22880008 PMC3411745

[B39] Isomäki P. Aversa G. Cocks B. G. Luukkainen R. Saario R. Toivanen P. (1997). Increased expression of signaling lymphocytic activation molecule in patients with rheumatoid arthritis and its role in the regulation of cytokine production in rheumatoid synovium. J. Immunol. 159 (6), 2986–2993. 10.4049/jimmunol.159.6.2986 9300723

[B40] Jang S. Kwon E. J. Lee J. J. (2022). Rheumatoid arthritis: pathogenic roles of diverse immune cells. Int. J. Mol. Sci. 23 (2), 905. 10.3390/ijms23020905 35055087 PMC8780115

[B41] Jiang Q. Yang G. Liu Q. Wang S. Cui D. (2021). Function and role of regulatory T cells in rheumatoid arthritis. Front. Immunol. 12, 626193. 10.3389/fimmu.2021.626193 33868244 PMC8047316

[B42] Kamradt T. Mitchison N. A. (2001). Tolerance and autoimmunity. N. Engl. J. Med. 344 (9), 655–664. 10.1056/nejm200103013440907 11228281

[B43] Karampetsou M. P. Comte D. Kis-Toth K. Kyttaris V. C. Tsokos G. C. (2017). Expression patterns of signaling lymphocytic activation molecule family members in peripheral blood mononuclear cell subsets in patients with systemic lupus erythematosus. PLoS One 12 (10), e0186073. 10.1371/journal.pone.0186073 29020082 PMC5636110

[B44] Kingsbury G. A. Feeney L. A. Nong Y. Calandra S. A. Murphy C. J. Corcoran J. M. (2001). Cloning, expression, and function of BLAME, a novel member of the CD2 family. J. Immunol. 166 (9), 5675–5680. 10.4049/jimmunol.166.9.5675 11313408

[B45] Kis-Toth K. Tsokos G. C. (2014). Engagement of SLAMF2/CD48 prolongs the time frame of effective T cell activation by supporting mature dendritic cell survival. J. Immunol. 192 (9), 4436–4442. 10.4049/jimmunol.1302909 24670806 PMC4017928

[B46] Korb-Pap A. Stratis A. Mühlenberg K. Niederreiter B. Hayer S. Echtermeyer F. (2012). Early structural changes in cartilage and bone are required for the attachment and invasion of inflamed synovial tissue during destructive inflammatory arthritis. Ann. Rheum. Dis. 71 (6), 1004–1011. 10.1136/annrheumdis-2011-200386 22258493

[B47] Kumaresan P. R. Mathew P. A. (2000). Structure of the human natural killer cell receptor 2B4 gene and identification of a novel alternative transcript. Immunogenetics 51 (11), 987–992. 10.1007/s002510000237 11003394

[B48] Kwon Y. C. Lim J. Bang S. Y. Ha E. Hwang M. Y. Yoon K. (2020). Genome-wide association study in a Korean population identifies six novel susceptibility loci for rheumatoid arthritis. Ann. Rheum. Dis. 79 (11), 1438–1445. 10.1136/annrheumdis-2020-217663 32723749 PMC7569386

[B49] Lee J. K. Mathew S. O. Vaidya S. V. Kumaresan P. R. Mathew P. A. (2007). CS1 (CRACC, CD319) induces proliferation and autocrine cytokine expression on human B lymphocytes. J. Immunol. 179 (7), 4672–4678. 10.4049/jimmunol.179.7.4672 17878365

[B50] Lenz T. L. Deutsch A. J. Han B. Hu X. Okada Y. Eyre S. (2015). Widespread non-additive and interaction effects within HLA loci modulate the risk of autoimmune diseases. Nat. Genet. 47 (9), 1085–1090. 10.1038/ng.3379 26258845 PMC4552599

[B51] Li A. Zhang Z. Ru X. Yi Y. Li X. Qian J. (2022). Identification of SLAMF1 as an immune-related key gene associated with rheumatoid arthritis and verified in mice collagen-induced arthritis model. Front. Immunol. 13, 961129. 10.3389/fimmu.2022.961129 36110846 PMC9468826

[B52] Liu J. Huang Y. Zeng J. Chen C. Li P. Ning Q. (2022). SLAMF8 promotes the proliferation and migration of synovial fibroblasts by regulating the ERK/MMPs signalling pathway. Autoimmunity 55 (5), 294–300. 10.1080/08916934.2022.2070742 35506438

[B53] Martin M. Romero X. de la Fuente M. A. Tovar V. Zapater N. R. Esplugues E. (2001). CD84 functions as a homophilic adhesion molecule and enhances IFN-γ secretion: adhesion is mediated by Ig-like domain 1. J. Immunol. 167 (7), 3668–3676. 10.4049/jimmunol.167.7.3668 11564780

[B54] Maruotti N. Annese T. Cantatore F. P. Ribatti D. (2013). Macrophages and angiogenesis in rheumatic diseases. Vasc. Cell. 5, 11–18. 10.1186/2045-824X-5-11 23725043 PMC3680215

[B55] Matsui Y. Shibano K. Kashiwagi H. Yamakawa-Kobayashi K. Inoko H. Staunton D. E. (1990). Restriction fragment length polymorphism of a lymphocyte surface antigen, Blast-1, in Japanese and Caucasians, and in patients with rheumatoid arthritis. Tissue Antigens 35 (5), 203–205. 10.1111/j.1399-0039.1990.tb01783.x 1976277

[B56] McArdel S. L. Terhorst C. Sharpe A. H. (2016). Roles of CD48 in regulating immunity and tolerance. Clin. Immunol. 164, 10–20. 10.1016/j.clim.2016.01.008 26794910 PMC4860950

[B57] Morita Y. Fukazawa T. Hirashima M. Kaga K. Kusaoi M. Morita T. (2006). The effect of methotrexate (MTX) on expression of signalling lymphocytic activation molecule (SLAM) in patients with rheumatoid arthritis (RA) and its role in the regulation of cytokine production. Scand. J. Rheumatol. 35 (4), 268–272. 10.1080/03009740600588186 16882589

[B58] Nanda N. Andre P. Bao M. Clauser K. Deguzman F. Howie D. (2005). Platelet aggregation induces platelet aggregate stability via SLAM family receptor signaling. Blood 106 (9), 3028–3034. 10.1182/blood-2005-01-0333 16037392

[B59] Nichols K. E. Hom J. Gong S. Y. Ganguly A. Ma C. S. Cannons J. L. (2005). Regulation of NKT cell development by SAP, the protein defective in XLP. Nat. Med. 11 (3), 340–345. 10.1038/nm1189 15711562 PMC10655637

[B60] Ohori M. (2008). ERK inhibitors as a potential new therapy for rheumatoid arthritis. Drug News Perspect. 21 (5), 245–250. 10.1358/dnp.2008.21.5.1219006 18596988

[B61] Okada Y. Wu D. Trynka G. Raj T. Terao C. Ikari K. (2014). Genetics of rheumatoid arthritis contributes to biology and drug discovery. Nature 506 (7488), 376–381. 10.1038/nature12873 24390342 PMC3944098

[B62] O'Keeffe M. S. Song J. H. Liao G. De Calisto J. Halibozek P. J. Mora J. R. (2015). SLAMF4 is a negative regulator of expansion of cytotoxic intraepithelial CD8+ T cells that maintains homeostasis in the small intestine. Gastroenterology 148 (5), 991–1001. 10.1053/j.gastro.2015.02.003 25678452 PMC4409516

[B63] Owczarczyk K. Lal P. Abbas A. R. Wolslegel K. Holweg C. T. Dummer W. (2011). A plasmablast biomarker for nonresponse to antibody therapy to CD20 in rheumatoid arthritis. Sci. Transl. Med. 3 (101), 101ra92. 10.1126/scitranslmed.3002432 21937757

[B64] Pereira M. Petretto E. Gordon S. Bassett J. D. Williams G. R. Behmoaras J. (2018). Common signalling pathways in macrophage and osteoclast multinucleation. J. Cell. Sci. 131 (11), jcs216267. 10.1242/jcs.216267 29871956

[B65] Punnonen J. Cocks B. G. Carballido J. M. Bennett B. Peterson D. Aversa G. (1997). Soluble and membrane-bound forms of signaling lymphocytic activation molecule (SLAM) induce proliferation and Ig synthesis by activated human B lymphocytes. J. Exp. Med. 185 (6), 993–1004. 10.1084/jem.185.6.993 9091591 PMC2196230

[B66] Qin W. Rong X. Yu C. Jia P. Yang J. Zhou G. (2022). Knockout of SLAMF8 attenuates collagen-induced rheumatoid arthritis in mice through inhibiting TLR4/NF-κB signaling pathway. Int. Immunopharmacol. 107, 108644. 10.1016/j.intimp.2022.108644 35259711

[B67] Rao D. A. Gurish M. F. Marshall J. L. Slowikowski K. Fonseka C. Y. Liu Y. (2017). Pathologically expanded peripheral T helper cell subset drives B cells in rheumatoid arthritis. Nature 542 (7639), 110–114. 10.1038/nature20810 28150777 PMC5349321

[B68] Réthi B. Gogolák P. Szatmari I. Veres A. Erdôs E. Nagy L. (2006). SLAM/SLAM interactions inhibit CD40-induced production of inflammatory cytokines in monocyte-derived dendritic cells. Blood 107 (7), 2821–2829. 10.1182/blood-2005-06-2265 16317102 PMC1895370

[B69] Rose B. J. Kooyman D. L. (2016). A tale of two joints: the role of matrix metalloproteases in cartilage biology. Dis. markers 2016, 4895050. 10.1155/2016/4895050 27478294 PMC4961809

[B70] Safiri S. Kolahi A. A. Hoy D. Smith E. Bettampadi D. Mansournia M. A. (2019). Global, regional and national burden of rheumatoid arthritis 1990-2017: a systematic analysis of the Global Burden of Disease study 2017. Ann. Rheum. Dis. 78 (11), 1463–1471. 10.1136/annrheumdis-2019-215920 31511227

[B71] Sandrin M. S. Henning M. M. Lo M. F. Baker E. Sutherland G. R. McKenzie I. F. (1996). Isolation and characterization of cDNA clones for Humly9: the human homologue of mouse Ly9. Immunogenetics 43 (1-2), 13–19. 10.1007/bf00186599 8537117

[B72] Shachar I. Barak A. Lewinsky H. Sever L. Radomir L. (2019). SLAMF receptors on normal and malignant B cells. Clin. Immunol. 204, 23–30. 10.1016/j.clim.2018.10.020 30448442

[B73] Simmons D. P. Nguyen H. N. Gomez-Rivas E. Jeong Y. Jonsson A. H. Chen A. F. (2022). SLAMF7 engagement superactivates macrophages in acute and chronic inflammation. Sci. Immunol. 7 (68), eabf2846. 10.1126/sciimmunol.abf2846 35148199 PMC8991457

[B74] Smolen J. S. Aletaha D. McInnes I. B. (2016). Rheumatoid arthritis. Lancet 388 (10055), 2023–2038. 10.1016/s0140-6736(16)30173-8 27156434

[B75] Staunton D. E. Fisher R. C. LeBeau M. M. Lawrence J. B. Barton D. E. Francke U. (1989). Blast-1 possesses a glycosyl-phosphatidylinositol (GPI) membrane anchor, is related to LFA-3 and OX-45, and maps to chromosome 1q21-23. J. Exp. Med. 169 (3), 1087–1099. 10.1084/jem.169.3.1087 2466936 PMC2189294

[B76] Sugimoto A. Kataoka T. R. Ito H. Kitamura K. Saito N. Hirata M. (2020). SLAM family member 8 is expressed in and enhances the growth of anaplastic large cell lymphoma. Sci. Rep. 10 (1), 2505. 10.1038/s41598-020-59530-1 32054954 PMC7018816

[B77] Sugimoto A. Kataoka T. R. Ueshima C. Takei Y. Kitamura K. Hirata M. (2018). SLAM family member 8 is involved in oncogenic KIT-mediated signalling in human mastocytosis. Exp. Dermatol 27 (6), 641–646. 10.1111/exd.13523 29498772

[B78] Sun J. Liu B. Yuan Y. Zhang L. Wang J. (2022). Disease markers and therapeutic targets for rheumatoid arthritis identified by integrating bioinformatics analysis with virtual screening of traditional Chinese medicine. Front. Biosci. (Landmark Ed. 27 (9), 267. 10.31083/j.fbl2709267 36224010

[B79] Suzuki A. Yamada R. Kochi Y. Sawada T. Okada Y. Matsuda K. (2008). Functional SNPs in CD244 increase the risk of rheumatoid arthritis in a Japanese population. Nat. Genet. 40 (10), 1224–1229. 10.1038/ng.205 18794858

[B80] Tuncer T. Kaya A. Gulkesen A. Kal G. A. Kaman D. Akgol G. (2019). Matrix metalloproteinase-3 levels in relation to disease activity and radiological progression in rheumatoid arthritis. Adv. Clin. Exp. Med. 28 (5), 665–670. 10.17219/acem/94065 30740946

[B81] van Driel B. J. Liao G. Engel P. Terhorst C. (2016). Responses to microbial challenges by SLAMF receptors. Front. Immunol. 7, 4. 10.3389/fimmu.2016.00004 26834746 PMC4718992

[B82] Veillette A. (2006). Immune regulation by SLAM family receptors and SAP-related adaptors. Nat. Rev. Immunol. 6 (1), 56–66. 10.1038/nri1761 16493427

[B83] Volkova O. Guselnikov S. Mechetina L. Chikaev N. Baranov K. Kulemzin S. (2014). Development and characterization of domain-specific monoclonal antibodies produced against human SLAMF9. Monoclon. Antib. Immunodiagn. Immunother. 33 (4), 209–214. 10.1089/mab.2014.0010 25170999 PMC4151055

[B84] Wang N. Halibozek P. J. Yigit B. Zhao H. O'Keeffe M. S. Sage P. (2015). Negative regulation of humoral immunity due to interplay between the SLAMF1, SLAMF5, and SLAMF6 receptors. Front. Immunol. 6, 158. 10.3389/fimmu.2015.00158 25926831 PMC4396446

[B85] Wang N. Satoskar A. Faubion W. Howie D. Okamoto S. Feske S. (2004). The cell surface receptor SLAM controls T cell and macrophage functions. J. Exp. Med. 199 (9), 1255–1264. 10.1084/jem.20031835 15123745 PMC2211908

[B86] Woo J. Vierboom M. P. Kwon H. Chao D. Ye S. Li J. (2013). PDL241, a novel humanized monoclonal antibody, reveals CD319 as a therapeutic target for rheumatoid arthritis. Arthritis Res. Ther. 15 (6), R207. 10.1186/ar4400 24299175 PMC3978732

[B87] Wu D. Zhang P. Ma J. Xu J. Yang L. Xu W. (2019). Serum biomarker panels for the diagnosis of gastric cancer. Cancer Med. 8 (4), 1576–1583. 10.1002/cam4.2055 30873760 PMC6488129

[B88] Xia G. Li Y. Pan W. Qian C. Ma L. Zhou J. (2022). SLAMF6 is associated with the susceptibility and severity of rheumatoid arthritis in the Chinese population. J. Orthop. Surg. Res. 17 (1), 13. 10.1186/s13018-021-02901-9 35016729 PMC8753921

[B89] Yan Q. Malashkevich V. N. Fedorov A. Fedorov E. Cao E. Lary J. W. (2007). Structure of CD84 provides insight into SLAM family function. Proc. Natl. Acad. Sci. U. S. A. 104 (25), 10583–10588. 10.1073/pnas.0703893104 17563375 PMC1965556

[B90] Zaiss M. Hirtreiter C. Rehli M. Rehm A. Kunz-Schughart L. A. Andreesen R. (2003). CD84 expression on human hematopoietic progenitor cells. Exp. Hematol. 31 (9), 798–805. 10.1016/s0301-472x(03)00187-5 12962726

[B91] Zhang Q. Cheng L. Qin Y. Kong L. Shi X. Hu J. (2021). SLAMF8 expression predicts the efficacy of anti-PD1 immunotherapy in gastrointestinal cancers. Clin. Transl. Immunol. 10 (10), e1347. 10.1002/cti2.1347 PMC8546794 34729183

[B92] Zhao J. Wei K. Chang C. Xu L. Jiang P. Guo S. (2022a). DNA methylation of T lymphocytes as a therapeutic target: implications for rheumatoid arthritis etiology. Front. Immunol. 13, 863703. 10.3389/fimmu.2022.863703 35309322 PMC8927780

[B93] Zhao J. Wei K. Jiang P. Chang C. Xu L. Xu L. (2022b). G-Protein-Coupled receptors in rheumatoid arthritis: recent insights into mechanisms and functional roles. Front. Immunol. 13, 907733. 10.3389/fimmu.2022.907733 35874704 PMC9304905

[B94] Zhao J. Xu L. Chang C. Jiang P. Wei K. Shi Y. (2022c). Circulating methylation level of HTR2A is associated with inflammation and disease activity in rheumatoid arthritis. Front. Immunol. 13, 1054451. 10.3389/fimmu.2022.1054451 36561742 PMC9763304

[B95] Zou C. Y. Guan G. F. Zhu C. Liu T. Q. Guo Q. Cheng W. (2019). Costimulatory checkpoint SLAMF8 is an independent prognosis factor in glioma. CNS Neurosci. Ther. 25 (3), 333–342. 10.1111/cns.13041 30105842 PMC6488888

